# (2,7-Dimeth­oxy­naphthalen-1-yl)(4-fluoro­phen­yl)methanone

**DOI:** 10.1107/S1600536811018332

**Published:** 2011-05-20

**Authors:** Shoji Watanabe, Toyokazu Muto, Atsushi Nagasawa, Akiko Okamoto, Noriyuki Yonezawa

**Affiliations:** a Department of Organic and Polymer Materials Chemistry, Tokyo University of Agriculture & Technology, Koganei, Tokyo 184-8588, Japan

## Abstract

In the title compound, C_19_H_15_FO_3_, the dihedral angle between the naphthalene ring system and the benzene ring is 80.46 (4)°. In the crystal, mol­ecules are linked by inter­molecular C—H⋯O hydrogen bonds into chains parallel to the *b* axis.

## Related literature

For the formation reaction of aroylated naphthalene compounds *via* electrophilic aromatic aroylation of 2,7-dimeth­oxy­naphthalene, see: Okamoto & Yonezawa (2009 ▶). For related structures reported by our group, see: Kato *et al.* (2010 ▶); Muto *et al.* (2010 ▶); Watanabe, Nagasawa *et al.* (2010 ▶); Watanabe, Nakaema, Muto *et al.* (2010 ▶); Watanabe, Nakaema, Nishijima *et al.* (2010 ▶).
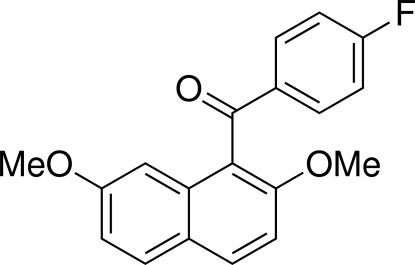


## Experimental

### 

#### Crystal data

C_19_H_15_FO_3_
*M* *_r_* = 310.31
Monoclinic, 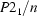

*a* = 10.9714 (2) Å
*b* = 7.51791 (14) Å
*c* = 18.7832 (3) Å
β = 99.917 (1)°
*V* = 1526.13 (5) Å^3^
*Z* = 4
Cu *K*α radiation
μ = 0.82 mm^−1^
*T* = 193 K
0.40 × 0.30 × 0.20 mm

#### Data collection

Rigaku R-AXIS RAPID diffractometer
Absorption correction: numerical (*NUMABS*; Higashi, 1999 ▶) *T*_min_ = 0.735, *T*_max_ = 0.853
26625 measured reflections
2789 independent reflections
2566 reflections with *I* > 2σ(*I*)
*R*_int_ = 0.036

#### Refinement

*R*[*F* ^2^ > 2σ(*F* ^2^)] = 0.034
*wR*(*F* ^2^) = 0.109
*S* = 1.01
2789 reflections
211 parameters
H-atom parameters constrained
Δρ_max_ = 0.23 e Å^−3^
Δρ_min_ = −0.15 e Å^−3^

### 

Data collection: *PROCESS-AUTO* (Rigaku, 1998 ▶); cell refinement: *PROCESS-AUTO*; data reduction: *CrystalStructure* (Rigaku/MSC, 2004 ▶); program(s) used to solve structure: *SIR2004* (Burla *et al.*, 2005 ▶); program(s) used to refine structure: *SHELXL97* (Sheldrick, 2008 ▶); molecular graphics: *ORTEP* (Burnett & Johnson, 1996 ▶); software used to prepare material for publication: *SHELXL97*.

## Supplementary Material

Crystal structure: contains datablocks I, global. DOI: 10.1107/S1600536811018332/rz2596sup1.cif

Structure factors: contains datablocks I. DOI: 10.1107/S1600536811018332/rz2596Isup2.hkl

Supplementary material file. DOI: 10.1107/S1600536811018332/rz2596Isup3.cml

Additional supplementary materials:  crystallographic information; 3D view; checkCIF report

## Figures and Tables

**Table 1:** Hydrogen-bond geometry (Å, °)

*D*—H⋯*A*	*D*—H	H⋯*A*	*D*⋯*A*	*D*—H⋯*A*
C14—H14⋯O1^i^	0.95	2.35	3.2139 (15)	151

Symmetry code: (i) 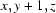.

**Fig. 1.**
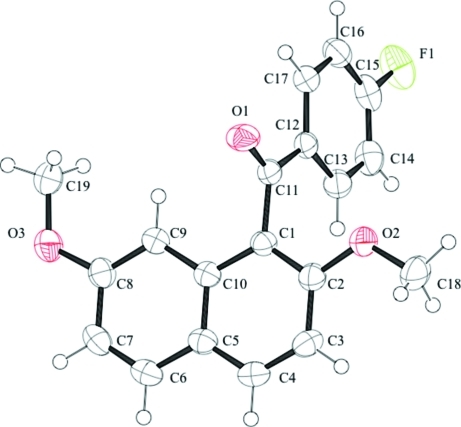
The molecular structure of (I), showing 50% probability displacement ellipsoids.

**Fig. 2.**
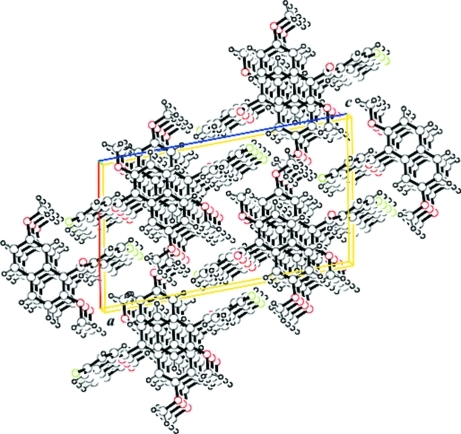
The alignment of the molecules in the crystal structure, viewed down the b axis.

**Fig. 3.**
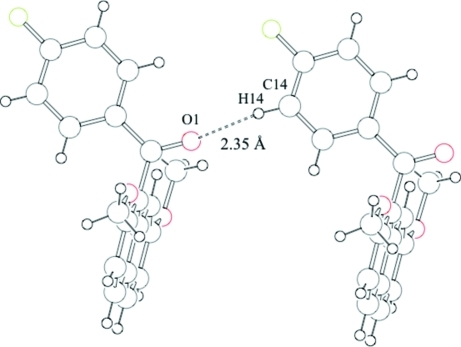
C–H···O interaction between hydrogen atom of fluorophenyl ring and carbonyl oxygen atom.

## supplementary crystallographic information

### Comment

In the course of our study on electrophilic aromatic aroylation of 2,7-dimethoxynaphthalene, *peri*-aroylnaphthalene compounds have proven to be formed regioselectively with the aid of suitable acidic mediators (Okamoto & Yonezawa, 2009). The aroyl groups at the 1,8-positions of the naphthalene rings in these compounds are twisted almost perpendicularly but the benzene ring moieties of the aroyl groups tilt slightly toward the *exo* sides of the naphthalene rings. Recently, we reported the structures of 1,8-diaroyl-2,7-dimethoxynaphthalenes, i. e., (2,7-dimethoxynaphthalene-1,8-diyl)bis(4-fluorophenyl)dimethanone (Watanabe, Nagasawa *et al.*, 2010), bis(4-bromophenyl)(2,7-dimethoxynaphthalene-1,8-diyl)dimethanone (Watanabe, Nakaema, Muto *et al.*, 2010), and [2,7-dimethoxy-8-(4-methylbenzoyl)-1-naphthyl](4-methylphenyl)methanone (Muto *et al.*, 2010). Furthermore, the crystal structures of 1-aroyl-2,7-dimethoxynaphthalenes, i. e., 2,7-dimethoxy-1-(4-nitrobenzoyl)naphthalene (Watanabe, Nakaema, Nishijima *et al.*, 2010) and (2,7-dimethoxynaphthalen-1-yl)(phenyl)methanone (Kato *et al.*, 2010), also exhibit essentially the same non-coplanar structure as the 1,8-diaroylated naphthalenes. As a part of our ongoing studies on the formation and the structure of the aroylated naphthalene derivatives, the synthesis and crystal structure of (I), a 1-monoaroylnaphthalene bearing fluoro group, is discussed in this report. (I) was prepared by electrophilic aromatic aroylation reaction of 2,7-dimethoxynaphthalene with 4-fluorobenzoyl chloride.

The molecular structure of (I) is displayed in Fig. 1. The interplanar angle between the benzene ring (C12—C17) and the naphthalene ring (C1—C10) is 80.46 (4)°. The torsion angle between the carbonyl group and the naphthalene ring [C10–C1–C11–O1 = -77.77 (13)°] is larger than that between the carbonyl group and fluorophenyl ring [O1–C11–C12–C17 = 4.20 (15)°].

In the crystal packing, the molecules are aligned consecutively in stacks along the *b* axis (Fig. 2). This stack of naphthalene rings occludes the adjacent counter part and *vice versa*. The crystal packing is stabilized by weak intermolecular C—H···O hydrogen bond between the hydrogen atom of the 4-fluorophenyl group and the carbonyl oxygen atom (Table 1; Fig. 3).

### Experimental

The title compound was prepared by treatment of a mixture of 2,7-dimethoxynaphthalene (75.29 mg, 0.4 mmol), 4-fluorobenzoyl chloride (69.77 mg, 0.44 mmol), CH_2_Cl_2_ (1 ml) with AlCl_3_ (0.48 mmol, 64.00 mg). After the reaction mixture was stirred at 273 K for 3 h, the mixture was poured into ice-cooled water and extracted with CHCl_3_ (10 ml × 3). The combined extracts were washed with 2 *M* aqueous NaOH followed by washing with brine. The organic layer thus obtained was dried over anhydrous MgSO_4_. The solvent was removed under reduced pressure to give cake. The crude product was purified by recrystallization from ethanol (isolated yield 76%). Colourless platelet single crystals suitable for X-ray analysis were obtained by slow evaporation of an ethanol solution (m.p. 381 K). Anal. Calcd for C_19_H_15_O_3_F: C, 73.54; H, 4.87. Found: C, 73.45; H, 4.83. Spectroscopic data:

^1^H NMR (300 MHz, CDCl_3_. p.p.m.) 3.67 (3*H*, s), 3.75 (3*H*, s), 6.79 (1*H*, d, *J* = 2.4 Hz), 6.70 (1*H*, dd, *J* = 9.0, 2.4 Hz), 7.07 (2*H*, dd, *J* = 9.0, 9.0 Hz), 7.12 (1*H*, d, *J* = 9.0 Hz), 7.69 (1*H*, d, *J* = 9.0 Hz), 7.83 (1*H*, d, *J* = 9.0 Hz), 7.87 (2*H*, dd, *J* = 5.7, 8.7 Hz);

^13^C NMR (75.0 MHz, CDCl_3_, p.p.m.); 55.2945, 56.4131, 102.1511, 110.2777, 115.7894 (*J*_C–F_ = 22.39 Hz), 117.2283, 121.4923, 124.5039, 129.8388, 131.2824, 132.3007 (*J*_C–F_ = 9.39 Hz), 133.0798, 134.6669 (*J*_C–F_ = 2.88 Hz), 155.0597, 159.0656, 166.0784 (*J*_C–F_ = 255.03 Hz), 196.5529;

IR (KBr, cm^-1^): 1662, 1627, 1597, 1513, 1279, 1242;

### Refinement

All the H atoms were found in difference maps and were subsequently refined as riding atoms, with C—H = 0.95 (aromatic) and 0.98 (methyl) Å, and *U*_iso_(H) = 1.2*U*_eq_(C).

### Crystal data

**Table:**

C_19_H_15_FO_3_	*F*(000) = 648
*M**_r_* = 310.31	*D*_x_ = 1.351 Mg m^−^^3^
Monoclinic, *P*2_1_/*n*	Melting point: 381 K
Hall symbol: -P 2yn	Cu *K*α radiation, λ = 1.54187 Å
*a* = 10.9714 (2) Å	Cell parameters from 24946 reflections
*b* = 7.51791 (14) Å	θ = 4.1–68.3°
*c* = 18.7832 (3) Å	µ = 0.82 mm^−^^1^
β = 99.917 (1)°	*T* = 193 K
*V* = 1526.13 (5) Å^3^	Platelet, colourless
*Z* = 4	0.40 × 0.30 × 0.20 mm

### Data collection

**Table:**

Rigaku R-AXIS RAPID diffractometer	2789 independent reflections
Radiation source: fine-focus sealed tube	2566 reflections with *I* > 2σ(*I*)
graphite	*R*_int_ = 0.036
Detector resolution: 10.00 pixels mm^-1^	θ_max_ = 68.3°, θ_min_ = 4.4°
ω scans	*h* = −13→13
Absorption correction: numerical (*NUMABS*; Higashi, 1999)	*k* = −9→9
*T*_min_ = 0.735, *T*_max_ = 0.853	*l* = −22→22
26625 measured reflections	

### Refinement

**Table:**

Refinement on *F*^2^	Secondary atom site location: difference Fourier map
Least-squares matrix: full	Hydrogen site location: inferred from neighbouring sites
*R*[*F*^2^ > 2σ(*F*^2^)] = 0.034	H-atom parameters constrained
*wR*(*F*^2^) = 0.109	*w* = 1/[σ^2^(*F*_o_^2^) + (0.0726*P*)^2^ + 0.253*P*] where *P* = (*F*_o_^2^ + 2*F*_c_^2^)/3
*S* = 1.01	(Δ/σ)_max_ = 0.001
2789 reflections	Δρ_max_ = 0.23 e Å^−^^3^
211 parameters	Δρ_min_ = −0.15 e Å^−^^3^
0 restraints	Extinction correction: *SHELXL97* (Sheldrick, 2008), Fc^*^=kFc[1+0.001xFc^2^λ^3^/sin(2θ)]^-1/4^
Primary atom site location: structure-invariant direct methods	Extinction coefficient: 0.0116 (8)

### Special details

**Table:**

Geometry. All e.s.d.'s (except the e.s.d. in the dihedral angle between two l.s. planes) are estimated using the full covariance matrix. The cell e.s.d.'s are taken into account individually in the estimation of e.s.d.'s in distances, angles and torsion angles; correlations between e.s.d.'s in cell parameters are only used when they are defined by crystal symmetry. An approximate (isotropic) treatment of cell e.s.d.'s is used for estimating e.s.d.'s involving l.s. planes.
Refinement. Refinement of *F*^2^ against ALL reflections. The weighted *R*-factor *wR* and goodness of fit *S* are based on *F*^2^, conventional *R*-factors *R* are based on *F*, with *F* set to zero for negative *F*^2^. The threshold expression of *F*^2^ > σ(*F*^2^) is used only for calculating *R*-factors(gt) *etc*. and is not relevant to the choice of reflections for refinement. *R*-factors based on *F*^2^ are statistically about twice as large as those based on *F*, and *R*- factors based on ALL data will be even larger.

### Fractional atomic coordinates and isotropic or equivalent isotropic displacement parameters (Å^2^)

**Table:**

	*x*	*y*	*z*	*U*_iso_*/*U*_eq_	
F1	0.88989 (8)	1.11551 (11)	0.39053 (4)	0.0625 (3)	
O1	0.83508 (8)	0.41722 (11)	0.56528 (4)	0.0452 (2)	
O2	0.56864 (7)	0.61906 (13)	0.58208 (4)	0.0467 (3)	
O3	1.18714 (7)	0.58367 (13)	0.78977 (5)	0.0485 (3)	
C1	0.76748 (10)	0.61523 (14)	0.64820 (6)	0.0316 (3)	
C2	0.64274 (10)	0.63620 (15)	0.64810 (6)	0.0366 (3)	
C3	0.59851 (10)	0.67163 (16)	0.71271 (6)	0.0407 (3)	
H3	0.5124	0.6863	0.7123	0.049*	
C4	0.68059 (11)	0.68475 (15)	0.77614 (6)	0.0391 (3)	
H4	0.6504	0.7093	0.8196	0.047*	
C5	0.80873 (10)	0.66282 (14)	0.77862 (6)	0.0345 (3)	
C6	0.89515 (11)	0.67527 (16)	0.84395 (6)	0.0403 (3)	
H6	0.8663	0.7023	0.8876	0.048*	
C7	1.01804 (11)	0.64943 (17)	0.84565 (6)	0.0426 (3)	
H7	1.0742	0.6570	0.8901	0.051*	
C8	1.06219 (10)	0.61110 (15)	0.78062 (6)	0.0374 (3)	
C9	0.98303 (10)	0.60226 (14)	0.71590 (6)	0.0332 (3)	
H9	1.0143	0.5797	0.6726	0.040*	
C10	0.85384 (10)	0.62683 (13)	0.71348 (5)	0.0312 (3)	
C11	0.81034 (9)	0.57083 (14)	0.57830 (5)	0.0317 (3)	
C12	0.82601 (9)	0.71631 (14)	0.52758 (5)	0.0315 (3)	
C13	0.79565 (10)	0.89121 (15)	0.54125 (6)	0.0391 (3)	
H13	0.7610	0.9179	0.5830	0.047*	
C14	0.81544 (11)	1.02663 (16)	0.49468 (7)	0.0450 (3)	
H14	0.7940	1.1461	0.5034	0.054*	
C15	0.86713 (11)	0.98285 (17)	0.43532 (6)	0.0435 (3)	
C16	0.89914 (10)	0.81228 (17)	0.42000 (6)	0.0425 (3)	
H16	0.9352	0.7874	0.3786	0.051*	
C17	0.87757 (10)	0.67828 (16)	0.46618 (6)	0.0367 (3)	
H17	0.8979	0.5590	0.4563	0.044*	
C18	0.43866 (11)	0.6376 (2)	0.57767 (8)	0.0608 (4)	
H18A	0.3977	0.6210	0.5275	0.073*	
H18B	0.4200	0.7567	0.5941	0.073*	
H18C	0.4087	0.5480	0.6084	0.073*	
C19	1.23927 (11)	0.5406 (2)	0.72769 (7)	0.0501 (3)	
H19A	1.3281	0.5187	0.7421	0.060*	
H19B	1.2264	0.6399	0.6934	0.060*	
H19C	1.1992	0.4336	0.7048	0.060*	

### Atomic displacement parameters (Å^2^)

**Table:**

	*U*^11^	*U*^22^	*U*^33^	*U*^12^	*U*^13^	*U*^23^
F1	0.0694 (5)	0.0562 (5)	0.0572 (5)	−0.0178 (4)	−0.0021 (4)	0.0216 (4)
O1	0.0652 (6)	0.0331 (5)	0.0399 (5)	0.0095 (4)	0.0167 (4)	−0.0010 (3)
O2	0.0323 (4)	0.0658 (6)	0.0400 (5)	0.0059 (4)	0.0003 (3)	−0.0088 (4)
O3	0.0354 (4)	0.0644 (6)	0.0426 (5)	−0.0001 (4)	−0.0025 (3)	−0.0037 (4)
C1	0.0346 (5)	0.0299 (5)	0.0307 (5)	0.0018 (4)	0.0066 (4)	−0.0017 (4)
C2	0.0355 (6)	0.0369 (6)	0.0367 (6)	0.0024 (4)	0.0038 (4)	−0.0040 (4)
C3	0.0346 (6)	0.0434 (6)	0.0464 (6)	0.0019 (5)	0.0133 (5)	−0.0059 (5)
C4	0.0453 (6)	0.0375 (6)	0.0375 (6)	0.0000 (5)	0.0154 (5)	−0.0052 (5)
C5	0.0423 (6)	0.0294 (5)	0.0326 (5)	−0.0016 (4)	0.0091 (4)	−0.0014 (4)
C6	0.0523 (7)	0.0396 (6)	0.0299 (5)	−0.0029 (5)	0.0092 (5)	−0.0032 (4)
C7	0.0487 (7)	0.0451 (7)	0.0307 (6)	−0.0029 (5)	−0.0027 (5)	−0.0017 (5)
C8	0.0362 (6)	0.0367 (6)	0.0379 (6)	−0.0031 (4)	0.0019 (4)	0.0004 (4)
C9	0.0355 (6)	0.0330 (6)	0.0313 (5)	−0.0018 (4)	0.0062 (4)	−0.0003 (4)
C10	0.0363 (6)	0.0262 (5)	0.0313 (5)	−0.0012 (4)	0.0064 (4)	−0.0004 (4)
C11	0.0297 (5)	0.0342 (6)	0.0299 (5)	0.0030 (4)	0.0013 (4)	−0.0033 (4)
C12	0.0281 (5)	0.0345 (6)	0.0300 (5)	0.0016 (4)	−0.0002 (4)	−0.0020 (4)
C13	0.0412 (6)	0.0367 (6)	0.0381 (6)	0.0033 (4)	0.0032 (5)	−0.0040 (5)
C14	0.0483 (7)	0.0324 (6)	0.0500 (7)	−0.0001 (5)	−0.0038 (5)	0.0006 (5)
C15	0.0399 (6)	0.0454 (7)	0.0407 (6)	−0.0092 (5)	−0.0061 (5)	0.0116 (5)
C16	0.0400 (6)	0.0542 (7)	0.0327 (6)	−0.0020 (5)	0.0044 (4)	0.0036 (5)
C17	0.0359 (5)	0.0410 (6)	0.0323 (5)	0.0044 (4)	0.0036 (4)	−0.0012 (4)
C18	0.0351 (7)	0.0828 (11)	0.0602 (8)	0.0114 (6)	−0.0034 (6)	−0.0184 (8)
C19	0.0356 (6)	0.0621 (8)	0.0513 (7)	0.0045 (5)	0.0038 (5)	−0.0006 (6)

### Geometric parameters (Å, °)

**Table:**

F1—C15	1.3554 (13)	C8—C9	1.3685 (15)
O1—C11	1.2207 (13)	C9—C10	1.4223 (15)
O2—C2	1.3668 (13)	C9—H9	0.9500
O2—C18	1.4210 (14)	C11—C12	1.4798 (15)
O3—C8	1.3676 (13)	C12—C13	1.3912 (15)
O3—C19	1.4217 (15)	C12—C17	1.3988 (15)
C1—C2	1.3773 (15)	C13—C14	1.3835 (17)
C1—C10	1.4173 (15)	C13—H13	0.9500
C1—C11	1.5063 (14)	C14—C15	1.3752 (18)
C2—C3	1.4078 (16)	C14—H14	0.9500
C3—C4	1.3676 (17)	C15—C16	1.3731 (18)
C3—H3	0.9500	C16—C17	1.3762 (17)
C4—C5	1.4083 (16)	C16—H16	0.9500
C4—H4	0.9500	C17—H17	0.9500
C5—C6	1.4188 (16)	C18—H18A	0.9800
C5—C10	1.4227 (15)	C18—H18B	0.9800
C6—C7	1.3572 (17)	C18—H18C	0.9800
C6—H6	0.9500	C19—H19A	0.9800
C7—C8	1.4189 (16)	C19—H19B	0.9800
C7—H7	0.9500	C19—H19C	0.9800
			
C2—O2—C18	118.54 (10)	O1—C11—C1	119.86 (9)
C8—O3—C19	117.86 (9)	C12—C11—C1	119.06 (9)
C2—C1—C10	120.68 (10)	C13—C12—C17	119.28 (10)
C2—C1—C11	118.92 (9)	C13—C12—C11	121.45 (10)
C10—C1—C11	120.34 (9)	C17—C12—C11	119.21 (10)
O2—C2—C1	115.21 (9)	C14—C13—C12	120.68 (11)
O2—C2—C3	124.09 (10)	C14—C13—H13	119.7
C1—C2—C3	120.70 (10)	C12—C13—H13	119.7
C4—C3—C2	119.44 (10)	C15—C14—C13	117.91 (11)
C4—C3—H3	120.3	C15—C14—H14	121.0
C2—C3—H3	120.3	C13—C14—H14	121.0
C3—C4—C5	121.61 (10)	F1—C15—C16	118.42 (11)
C3—C4—H4	119.2	F1—C15—C14	118.23 (12)
C5—C4—H4	119.2	C16—C15—C14	123.33 (11)
C4—C5—C6	122.38 (10)	C15—C16—C17	118.27 (11)
C4—C5—C10	119.14 (10)	C15—C16—H16	120.9
C6—C5—C10	118.48 (10)	C17—C16—H16	120.9
C7—C6—C5	121.60 (10)	C16—C17—C12	120.52 (11)
C7—C6—H6	119.2	C16—C17—H17	119.7
C5—C6—H6	119.2	C12—C17—H17	119.7
C6—C7—C8	119.60 (10)	O2—C18—H18A	109.5
C6—C7—H7	120.2	O2—C18—H18B	109.5
C8—C7—H7	120.2	H18A—C18—H18B	109.5
O3—C8—C9	124.99 (10)	O2—C18—H18C	109.5
O3—C8—C7	113.94 (10)	H18A—C18—H18C	109.5
C9—C8—C7	121.07 (10)	H18B—C18—H18C	109.5
C8—C9—C10	119.91 (10)	O3—C19—H19A	109.5
C8—C9—H9	120.0	O3—C19—H19B	109.5
C10—C9—H9	120.0	H19A—C19—H19B	109.5
C1—C10—C5	118.42 (10)	O3—C19—H19C	109.5
C1—C10—C9	122.26 (9)	H19A—C19—H19C	109.5
C5—C10—C9	119.31 (10)	H19B—C19—H19C	109.5
O1—C11—C12	121.05 (9)		
			
C18—O2—C2—C1	179.54 (11)	C4—C5—C10—C1	0.29 (15)
C18—O2—C2—C3	−0.10 (18)	C6—C5—C10—C1	−179.72 (10)
C10—C1—C2—O2	−178.91 (9)	C4—C5—C10—C9	−179.17 (10)
C11—C1—C2—O2	−1.87 (15)	C6—C5—C10—C9	0.82 (15)
C10—C1—C2—C3	0.74 (17)	C8—C9—C10—C1	−178.60 (10)
C11—C1—C2—C3	177.78 (10)	C8—C9—C10—C5	0.85 (15)
O2—C2—C3—C4	179.43 (11)	C2—C1—C11—O1	−99.28 (12)
C1—C2—C3—C4	−0.19 (18)	C10—C1—C11—O1	77.78 (13)
C2—C3—C4—C5	−0.31 (18)	C2—C1—C11—C12	82.89 (13)
C3—C4—C5—C6	−179.74 (11)	C10—C1—C11—C12	−100.06 (11)
C3—C4—C5—C10	0.25 (17)	O1—C11—C12—C13	178.65 (10)
C4—C5—C6—C7	178.35 (11)	C1—C11—C12—C13	−3.54 (14)
C10—C5—C6—C7	−1.64 (17)	O1—C11—C12—C17	−4.20 (15)
C5—C6—C7—C8	0.78 (18)	C1—C11—C12—C17	173.60 (9)
C19—O3—C8—C9	−0.77 (18)	C17—C12—C13—C14	0.30 (16)
C19—O3—C8—C7	178.67 (11)	C11—C12—C13—C14	177.44 (10)
C6—C7—C8—O3	−178.50 (11)	C12—C13—C14—C15	−0.74 (17)
C6—C7—C8—C9	0.96 (18)	C13—C14—C15—F1	−178.23 (10)
O3—C8—C9—C10	177.64 (10)	C13—C14—C15—C16	0.37 (17)
C7—C8—C9—C10	−1.76 (17)	F1—C15—C16—C17	179.06 (10)
C2—C1—C10—C5	−0.78 (16)	C14—C15—C16—C17	0.45 (17)
C11—C1—C10—C5	−177.78 (9)	C15—C16—C17—C12	−0.91 (16)
C2—C1—C10—C9	178.67 (10)	C13—C12—C17—C16	0.55 (15)
C11—C1—C10—C9	1.66 (16)	C11—C12—C17—C16	−176.65 (9)

### Hydrogen-bond geometry (Å, °)

**Table:**

*D*—H···*A*	*D*—H	H···*A*	*D*···*A*	*D*—H···*A*
C14—H14···O1^i^	0.95	2.35	3.2139 (15)	151

Symmetry codes: (i) *x*, *y*+1, *z*.
